# The effect of metabolic risk factors on urinary stone composition: An observational study

**DOI:** 10.1097/MD.0000000000029622

**Published:** 2022-07-15

**Authors:** Jae Yoon Kim, Ji Hyeong Yu, Seok Ho Kang, Jeong Gu Lee, Jun Cheon, Sung Gu Kang

**Affiliations:** a Department of Urology, Sanggye Paik Hospital, Inje University College of Medicine, Seoul, Republic of Korea; b Department of Urology, Korea University College of Medicine, Seoul, Republic of Korea.

**Keywords:** diabetes mellitus, metabolic disease, uric acid stone, urinary stone

## Abstract

To investigate how the risk factors of metabolic diseases affect urinary stone composition, particularly uric acid (UA) stones.

Overall, 583 patients with data on urinary stone composition were retrospectively analyzed and classified into UA and nonUA stone formers according to the presence of the UA component. Various factors were compared between both groups. Participants were categorized according to age, glucose level, HbA1c level, and estimated glomerular filtration rate (eGFR) into subgroups, and the incidence of UA stone was compared.

Overall, 137 UA stone formers (23.5%) and 446 nonUA stone formers (76.5%) were included. Mean age and male-to-female ratio were higher in the UA group than in the nonUA group. The rates of diabetes mellitus (DM), hypertension, chronic kidney disease, and coronary artery disease, all of which were associated with differences in urinary stone composition, were higher in the UA group than in the nonUA group. The UA group exhibited lower mean eGFR and higher glucose and HbA1c levels. Similarly, the UA group had higher mean UA levels and predictably lower urinary pH. In subgroup analysis, higher age, glucose level, HbA1c level, and lower eGFR were associated with an increased risk of UA stone formation. In the multivariate logistic regression analysis, the UA group showed a significantly higher age (*P* < .001), DM frequency (*P* = .049), and HbA1c level (*P* = .032), but significantly lower eGFR than the nonUA group (*P* < .001).

Age and DM were independent risk factors for UA urolithiasis, implying a relationship between urinary stone composition and metabolic diseases. Additionally, renal function and HbA1c level were risk factors for UA stones.

## 1. Introduction

Urolithiasis is a morbid condition whose prevalence rate has increased by 70% since 1994. Several risk factors have been shown to be associated with this detrimental and painful disease^[1]^; dietary tendencies, geographical conditions, racial traits, and weather are known risk factors for urolithiasis.^[2]^ The recurrence rate of urolithiasis is high (30%–50%) after initial stone elimination; therefore, preventing stone recurrence is extremely important.^[3]^

Recently, metabolic diseases have been emphasized to account for the increased incidence rate of urolithiasis.^[4]^ Westernized dietary habits and lifestyle changes play important roles in the increased incidence of metabolic syndrome, and there is growing evidence of the association between urinary stone formation and metabolic diseases or factors related to such conditions. A cross-sectional study reported an association between urolithiasis and metabolic syndrome.^[5]^ Furthermore, another large epidemiological study revealed that obesity, weight gain, hypertension, and diabetes mellitus (DM) were associated with an increased risk of nephrolithiasis.^[6]^

Several studies have reported that the effect of these metabolic risk factors is commonly associated with uric acid (UA) nephrolithiasis in the Western population.^[7–10]^ According to the European Association of Urology guidelines, all UA stone formers are at high risk of developing stone recurrence.^[11]^

The prevalence of UA stones shows global diversity, with the highest in the Middle East and a few European countries. The incidence of UA stones is 28% in Pakistan and 22% in Israel; in contrast, UA stones account for only 8%–10% of urinary stones in the United States, reflecting regional variations in climate, diet, and ethnicity.^[12]^ Furthermore, the high incidence of UA stones in China may be related to the local diet, which comprises animal protein and high alcohol intake.^[13]^ Nevertheless, the exact reason for the global diversity in the prevalence of UA stones remains unclear.

Therefore, this study aimed to investigate the effects of risk factors for metabolic diseases on urinary stone composition, specifically UA components, to facilitate a tailored metabolic assessment and treatment plan and prevent the recurrence of urinary stones.

## 2. Material and methods

### 2.1. Patients

**Figure 1.**
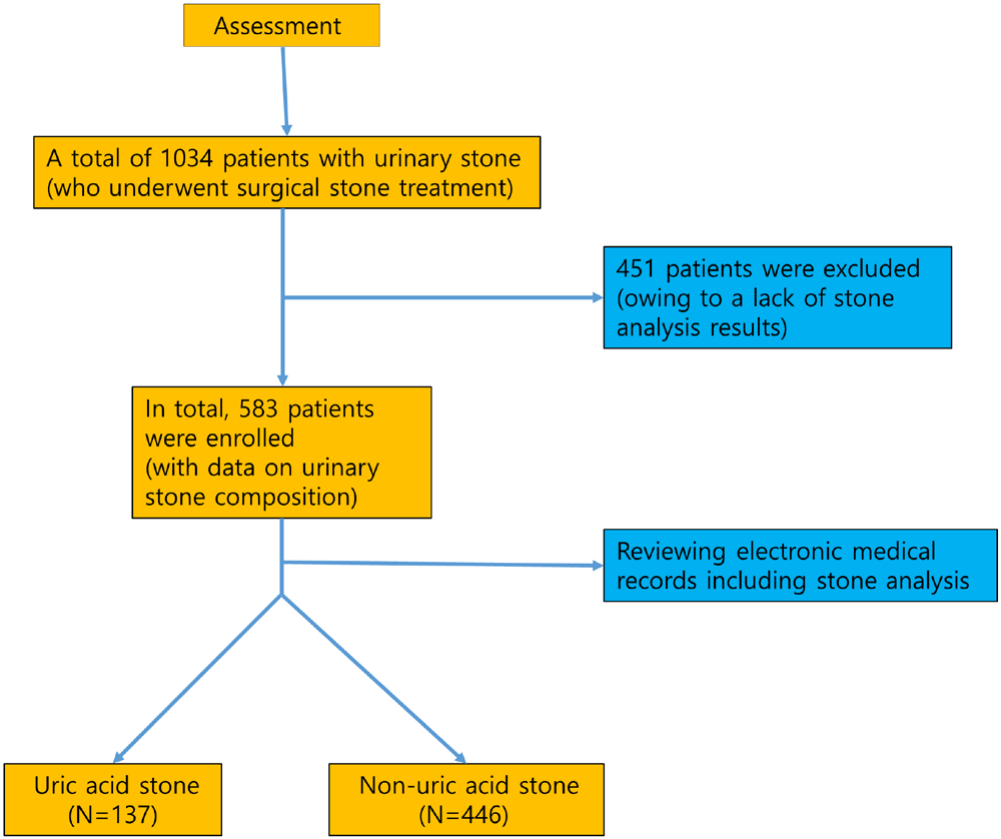
Flow chart of study participants.

Among 1034 patients who underwent surgical interventions (e.g., ureteroscopic lithotripsy, percutaneous nephrolithotomy, and laparoscopic ureterolithotomy) at a single center between March 2014 and May 2017, 451 were excluded from this study owing to a lack of stone analysis results. Finally, 583 patients with data on urinary stone composition were analyzed consecutively by retrospectively reviewing their electronic medical records, which were the source of all the data used in this study. Figure 1 illustrates the flow chart of study participants. The included subjects had never undergone any treatment for urinary stones previously. Patient data sources included clinical notes of medical history, medication lists, and laboratory and radiological results. Stone analysis was performed on samples obtained during the interventional procedure using infrared spectrometry. All obtained stone materials were sent for analysis to minimize sampling error according to the routine protocol at our institution.

The stone composition was classified according to the Mayo Clinic stone classification and the European Association of Urology guidelines.^[11,14]^ Patients were classified into UA and nonUA stone formers according to the presence of the UA component. UA stone formers comprised patients with any UA or urates as a component of urinary stone composition after surgery. Stones containing struvite, brushite, and cysteine were placed in the corresponding named groups. Stones composed of >50% calcium oxalate (CaOx) were classified as CaOx, whereas stones composed of >50% carbapatite were classified as carbapatites.

### 2.2. Main outcome measures

We compared the following variables between the UA and nonUA groups: age, sex, height, body weight, and body mass index (BMI); underlying diseases including DM, hyperlipidemia, hypertension, coronary artery disease (CAD), and chronic kidney disease (CKD); serum analysis results including estimated glomerular filtration rate (eGFR), glucose level, lipid profile (cholesterol, triglyceride, and high-density and low-density lipoprotein cholesterol levels), and glycosylated hemoglobin (HbA1c) level, which reflects metabolic status; and radiological results.

### 2.3. Subgroup analysis

We used the Modification of Diet in Renal Disease equation to determine the eGFR according to serum creatinine levels. The participants were categorized into the following 4 groups according to their baseline eGFR: group I, eGFR >90 mL/min/1.73 m^2^; group II, eGFR of 60 to 89 mL/min/1.73 m^2^; group III, eGFR of 30 to 59 mL/min/1.73 m^2^; and group IV, eGFR <30 mL/min/1.73 m^2^. The highest eGFR within 3 months of surgery was analyzed to prevent bias due to a temporary decrease in renal function caused by urinary obstruction and perioperative fluctuations.

We categorized the patients into several groups according to their age (<30, 30–50, 50 to 70, >70 years), glucose level (<100, 100–125, ≥126 mg/dL), and HbA1c level (<5.5%, 5.5 to 6.0%, 6.0–6.5%, ≥6.5%) to compare the incidence rate of UA stones among categorized groups using logistic regression analysis. Additionally, we compared parameters, including underlying diseases and laboratory results, according to urinary stone composition (UA, CaOx, carbapatite, struvite, brushite, and cysteine).

### 2.4. Statistical analysis

Sample size calculations were performed according to previous studies investigating the effect of metabolic risk factors on urinary stone composition. Assuming a similar treatment effect, a log-rank test with a 2-sided significance level of 0.05 had an 80% power to detect differences between the study groups, with a sample size of 123 in each group (at a ratio of 1:1). After considering a maximum drop-out rate of 10%, we included 137 patients in the UA stone group and 446 patients in the nonUA stone group, which comprised a larger population for a more accurate analysis.

Results are presented as proportions, means, and standard deviations. We used the *t*-test to analyze continuous variables, while the chi-square test and Fisher exact test were used to evaluate categorical variables with a 2-sided test as the statistical method. Additionally, the parameters were compared according to urinary stone composition using analysis of variance, followed by post hoc analysis with the Student-Newman-Keuls test. Multivariate logistic regression analysis was performed to determine factors related to UA stone formation by calculating odds ratios (ORs) and 95% confidence intervals (CIs).

Moreover, to adequately match the UA and nonUA groups, we performed a propensity score matched-pair analysis. Using DM, which is an important metabolic risk factor, as a classification variable, patients with DM were correspondingly matched with those without DM, considering the baseline clinical characteristics, including gender, age, height, and body weight.

Statistical analysis was performed using SPSS version 22.0 (IBM Corp., Armonk, NY), with the significance level set at *P* < .05.

### 2.5. Ethical approval

This study was approved by the Institutional Review Board (IRB) of Sanggye Paik Hospital, Inje University (committee reference number: 2021-06-006). All patients provided written informed consent.

## 3. Results

### 3.1. Patient characteristics

Overall, 583 patients with data on urinary stone composition were analyzed and classified into the UA (n = 137, 23.5%) and nonUA (n = 446, 76.5%) groups (Table 1). The mean age and male-to-female ratio were higher in the UA group than in the nonUA group (*P* < .001 and *P* < .001, respectively). Bodyweight and BMI showed no significant differences between the UA and nonUA groups (*P* = .251 and *P* = .676, respectively).

**Table 1:** Comparison of patient characteristics, underlying diseases, laboratory results, and radiologic findings between the UA and nonUA groups.

	UA stone	nonUA stone	*P*-value
Patient characteristics			
Patient (n)	137 (23.5%)	446 (76.5%)	
Male/female (n)	113/24 (82.5%/17.5%)	281/165 (63%/37%)	<.001
Age	65.5 ± 11.96	56.3 ± 13.84	<.001
Height	164.2 ± 8.50	162.8 ± 9.07	0.126
Body weight (kg)	67.6 ± 11.58	66.2 ± 12.22	0.251
BMI	25.0 ± 3.20	24.9 ± 3.43	0.676
Underlying disease (n)			
DM	41 (29.9%)	76 (17.0%)	**0.001**
Metformin use	20 (14.6%)	40 (9.0%)	0.058
Hyperlipidemia	20 (14.6%)	62 (13.9%)	0.838
Statin use	29 (13.9%)	60 (13.5%)	0.901
Hypertension	71 (51.8%)	176 (39.5%)	**0.011**
CKD	20 (14.6%)	17 (3.8%)	<.001
CAD	18 (13.1%)	28 (6.3%)	**0.026**
Laboratory test			
Serum eGFR	62.75 ± 30.03	92.50 ± 31.29	<.001
Calcium	8.97 ± 0.66	9.17 ± 0.63	**0.004**
Sodium	139.3 ± 2.73	139.3 ± 2.89	0.966
Cholesterol	172.4 ± 44.73	179.5 ± 40.62	0.087
Triglyceride	129.6 ± 80.25	141.5 ± 72.05	0.34
HDL	42.3 ± 11.30	46.0 ± 14.03	0.136
LDL	103.8 ± 35.16	112.7 ± 38.61	0.196
Glucose	135.3 ± 73.14	117.5 ± 38.01	**0.018**
HbA1c	6.66 ± 1.29	6.11 ± 1.18	**0.002**
UA	6.69 ± 2.03	5.41 ± 1.61	<.001
Urine pH	5.56 ± 0.69	6.08 ± 0.91	<.001
UTI (N)	31 (22.6%)	58 (13.1%)	**0.006**
Radiologic findings			
Stone size	5.88 ± 8.52	6.35 ± 9.21	0.61
Stone localization			**0.025**
Kidney	23.30%	33.20%	-
Ureter	47.40%	46.40%	0.184
Bladder	29.30%	20.40%	**0.026**
Hydronephrosis			0.075
HU	592 ± 310.2	1105 ± 374.7	<.001

Using the chi-square test, Fisher exact test, or *t*-test.

BMI = body mass index, CAD = coronary artery disease, CKD = chronic kidney disease, DM = diabetes mellitus, eGFR = estimated glomerular filtration rate, HbA1c = glycosylated hemoglobin, HDL = high-density lipoprotein, HU = Hounsfield unit, LDL = low-density lipoprotein, UA = uric acid, UTI = urinary tract infection.

### 3.2. Effects of the risk factors of metabolic diseases on urinary stone composition

Results for the association between metabolic parameters, including metabolic diseases, serum analysis results, and urinary stone composition, are summarized in Table 1. The dependent variable was urinary stone composition (reported as UA or nonUA stone), whereas the independent variables were metabolic risk factors. Chi-square analysis revealed that the proportions of patients with DM (29.9% vs 17.0%, *P* = 0.001), hypertension (51.8% vs 39.5%, *P* = .011), CKD (14.6% vs 3.8%, *P <* .001), and CAD (13.1% vs 6.3%, *P* = .026) were higher in the UA group than in the nonUA group, and all of these aforementioned variables were associated with differences in urinary stone composition (Table 1). Moreover, the UA group exhibited lower mean eGFR and calcium levels (*P <* .001 and *P* = .004, respectively) but higher glucose and HbA1c levels (*P* = .018 and *P* = .002, respectively) than the nonUA group. The UA group had higher mean UA levels and predictably lower urinary pH levels (*P <* .001 and *P <* .001, respectively). The lipid profile was not significantly different between both groups. Additionally, UA stones more frequently developed in the bladder than in the kidney in the nonUA group (29.3% vs 20.4%, *P* = .026). There was no difference in stone composition between kidney and ureter stones (Table 1).

### 3.3. Urinary stone composition

We presented the results comparing the parameters, including underlying diseases and laboratory results, according to urinary stone composition. Carbapatites were more frequently observed in female patients and those without metabolic diseases. Additionally, the mean age of patients with carbapatite stones was lower than that of the other groups. Struvite was observed more frequently in older and female patients, whereas the incidence of cysteine stones decreased with age. Otherwise, there was no significant difference among the other stone composition groups, except in the UA group (Table 1, Supplementary Digital Content, http://links.lww.com/MD/G844).

### 3.4. Subgroup analysis

**Figure 2.**
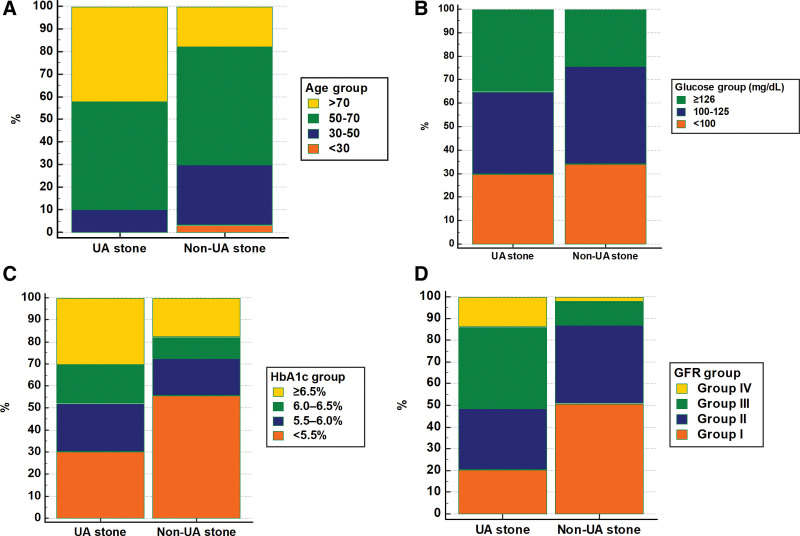
Subgroups categorized by age (A), glucose level (B), HbA1c level (C), and eGFR (D) in the UA and nonUA stone formers. eGFR = estimated glomerular filtration rate, HbA1c = glycosylated hemoglobin, UA = uric acid.

The incidence of UA stones was significantly different among the categorized age, glucose level, HbA1c level, and eGFR subgroups (*P* < .001, *P* = .046, *P* = .004, and *P* < .001, respectively). Higher age, glucose level, HbA1c level, and lower eGFR were associated with an increased risk of UA stone formation (Fig. 2).

Logistic regression analysis revealed that UA stone formation was significantly increased in patients aged >70 years (OR: 10.15, 95% CI: 1.298–79.379) than in those aged <30 years. Patients with glucose levels of ≥126 mg/dL had a significantly increased incidence of UA stones compared to those with glucose levels of <100 mg/dL (OR: 1.66, 95% CI: 1.014–2.726). The ORs for UA stone formation in patients with HbA1c levels of 5.5% to 6.0%, 6.0% to 6.5%, and ≥6.5% were 2.40 (95% CI: 1.09–5.26), 3.36 (95% CI: 1.39–8.14), and 3.13 (95% CI: 1.51–6.51), respectively, compared to that of those in patients with HbA1c levels of <5.5%. The corresponding ORs for UA stone formation in the eGFR groups II, III, and IV were 1.94 (95% CI: 1.15–3.29), 8.58 (95% CI: 4.92–14.96), and 17.04 (95% CI: 7.03–41.29), respectively, compared to those in eGFR group I (Table 2).

**Table 2:** Comparison of subgroups categorized by age, glucose level, HbA1c level, and eGFR between UA and nonUA stone formers.

	UA stone	nonUA stone	*P*-value	Hazard ratio (95% confidence interval)
Age (n)	0 (0%)	15 (3.4%)	<.001	–
<30	14 (10.2%)	119 (26.7%)	–	1.647 (0.201–13.49)
30–50	65 (47.4%)	232 (52.0%)	0.642	3.922 (0.506–30.39)
50–70	58 (42.3%)	80 (17.9%)	0.191	**10.15 (1.298–79.38**)
>70			**0.027**	
Glucose (mg/dL)	39 (29.8%)	148 (34.1%)	**0.046**	–
<100	46 (35.1%)	181 (41.7%)	–	0.964 (0.598–1.557)
100–125	46 (35.1%)	105 (24.2%)	0.882	**1.663 (1.014–2.726**)
≥126			**0.044**	
HbA1c	21 (30.4%)	94 (56.0%)	**0.004**	–
<5.5%	15 (21.7%)	28 (16.7%)	–	**2.398 (1.093–5.260**)
5.5–6.0%	12 (17.4%)	16 (9.5%)	**0.029**	**3.357 (1.385–8.138**)
6.0–6.5%	21 (30.4%)	30 (17.9%)	**0.007**	**3.133 (1.508–6.510**)
≥6.5%			**0.002**	
eGFR	28 (20.4%)	226 (50.8%)	<.001	–
Group I	39 (28.5%)	162 (36.4%)	–	**1.943 (1.149–3.287**)
Group II			**0.013**	
Group III	51 (37.2%)	48 (10.8%)	<.001	**8.576 (4.916–14.96**)
Group IV	19 (13.9%)	9 (2.0%)	<.001	**17.04 (7.033–41.29**)

Logistic regression analysis: Group I: eGFR >90 mL/min; group II: eGFR of 60–89 mL/min; group III: eGFR of 30–59 mL/min; group IV: eGFR <30 mL/min.

eGFR = estimated glomerular filtration rate, HbA1c = glycosylated hemoglobin, UA = uric acid.

On multivariate logistic regression analysis, age (95% CI: 1.083–1.839, *P* < .001) and the proportion of patients with DM (95% CI: 1.002–2.702, *P* = .049) were significantly higher in the UA group than in the nonUA group. Additionally, the UA group had a significantly higher HbA1c level (95% CI: 1.028–1.845, *P* = .032) but significantly lower eGFR than the nonUA group (95% CI: 0.964–0.982, *P* < .001) (Table 3). However, urinary stone composition showed no significant association with obesity index or dyslipidemia in our study.

**Table 3:** Multivariate logistic regression analysis of the effects of risk factors on UA stone formation.

	*P*-value	Hazard ratio	95% Confidence interval
DM	**0.049**	1.645	1.002–2.702
HbA1c	**0.032**	1.377	1.028–1.845
Age	<.001	1.411	1.083–1.839
eGFR	<.001	0.973	0.964–0.982

DM = diabetes mellitus, eGFR = estimated glomerular filtration rate, HbA1c = glycosylated hemoglobin.

Additionally, after the propensity score matched-pair analysis, 99 patients with DM were matched to 99 patients without DM, and both groups were comparable in terms of gender, age, height, and body weight (Table 4). In the matched-pair analysis, patients with DM showed a higher incidence of UA stone than those without DM (P = .029).

**Table 4:** Patient clinical characteristics stratified by the prevalence of DM after propensity score matched-pair analysis.

	Patients with DM (N = 99)	Patients without DM (N = 99)	*P*-value
Male/female (N)	71/28	71/28	1
	(71.7%/28.3%)	(71.7%/28.3%)	
Age	61.47 ± 11.53	61.4 ± 11.72	0.745
Height	163.4 ± 7.54	163.5 ± 7.11	0.896
Body weight (kg)	67.6 ± 10.87	67.5 ± 9.81	0.813
UA stone/nonUAstone (N)	34/65	20/79	**0.029**
	(34.3%/65.7%)	(20.2%/79.8%)	

DM = Diabetes mellitus, UA = Uric acid.

## 4. Discussion

Metabolic diseases were associated with differences in urinary stone composition. Age and DM were independent risk factors for UA urolithiasis, implying their association with urinary stone composition. Furthermore, renal function and HbA1c level were risk factors for UA stones, which are known to reflect metabolic status.

Several epidemiologic studies revealed the relationship between nephrolithiasis and numerous metabolic diseases, including DM, obesity, dyslipidemia, and hypertension. A large cross-sectional study reported that the prevalence of kidney stones increased with an increasing number of metabolic syndrome features, from 3% in the absence of features to 7.5% and 9.8% with 3 and 5 features, respectively.^[5]^ Furthermore, Jeong et al showed that metabolic syndrome status and the number of metabolic syndrome features were associated with an increased risk of kidney stones identified using computed tomography or ultrasonography in 34,895 individuals who underwent general health screening examinations.^[15]^ Kohjimoto et al suggested a significant correlation between the number of metabolic syndrome traits (obesity, DM, hypertension, and dyslipidemia) and the severity of kidney stone disease. In a large study evaluating 11,555 patients in Japan, the ORs were 1.8-fold greater in patients with 4 metabolic syndrome traits than in patients with no traits (OR: 1.78, 95% CI: 1.22–2.66).^[16]^

In this study, the prevalence of DM, hypertension, and CAD, which are components of metabolic syndrome, was significantly higher in the UA group than in the nonUA group. Adam et al showed that hypertension and DM were metabolic syndrome components independently associated with differences in urinary stone composition, specifically UA components.^[7]^ Other studies reported that the effects of metabolic syndrome on urinary stone composition are mainly in the area of UA stone diseases.^[8–10]^

The underlying causality behind this relationship remains unclear; nevertheless, several studies in the last decade have reported that the underlying pathway of nephrolithiasis in patients with metabolic diseases is associated with an increased risk of urinary derangements, including hypocitraturia, hypercalciuria, hyperuricosuria, hyperoxaluria, and especially low urinary pH levels. Furthermore, the mechanism underlying this increased risk of urolithiasis with DM might be attributed to higher acid excretion, impaired ammoniagenesis, and a subsequent decrease in urinary pH level resulting from insulin resistance.^[17,18]^ The precise mechanisms of reduced urinary excretion of ammonia and increased acid excretion have not been clearly demonstrated. Pollak et al suggested a defect in the enzymes, glutaminase and glutamate dehydrogenase, which metabolize glutamine to ammonia and α-ketoglutarate.^[19]^ Other investigators have demonstrated that the increased acid excretion is caused by organic acids produced by intestinal microflora.^[12]^ The UA stone type is dependent on the urinary pH level because its crystallization relies on urine acidity. Therefore, this alteration in urine can increase the incidence of urinary calculi, particularly UA stones. Similarly, Abate et al proposed a causative correlation between insulin resistance, low urinary ammonium levels, and low urinary pH levels in UA stone formers.^[8]^

Additionally, we found that the eGFR was significantly lower in the UA group than in the nonUA group. Furthermore, the group with lower eGFR (eGFR group IV) was associated with a higher risk of UA stone formation compared with the group with higher eGFR (eGFR group I) in the subgroup analysis. Adam et al suggested that lower urinary pH in patients is associated with impaired renal function.^[20]^ Renal stone composition differed significantly according to eGFR, with UA stones associated with a lower eGFR. In another study, Patel et al showed the association between decreased renal function and UA stones, lower urinary pH, lower calcium excretion, and greater oxalate excretion. In their study, UA stones appeared to be associated with decreased renal function, with the highest rate of UA stone composition observed in severe CKD.^[21]^ These previous reports suggest that renal function is an underestimated risk factor for UA stone formation. Additionally, these patients may harbor other metabolic risk factors, such as insulin resistance, which predispose them to UA stones.

Several studies have reported that more severe DM is associated with a higher risk of urolithiasis. Weinberg et al performed a study on 12,110 participants of the National Health and Nutrition Examination Survey and reported an increased incidence of kidney stone disease in patients with fasting plasma glucose levels of 100 to 126 mg/dL and >126 mg/dL. The corresponding ORs for patients with an HbA1c level of 5.7 to 6.4% and >6.5% were 1.68 (95% CI: 1.17–2.42) and 2.82 (95% CI: 1.98–4.02), respectively.^[22]^ Kabeya et al reported similar results. In their study, fasting plasma glucose and HbA1c levels were significantly associated with the risk of urolithiasis. Patients with fasting plasma glucose levels ≥126 mg/dL had an OR of 1.83 (95% CI: 0.7–2.23) compared to those with fasting plasma glucose levels <100 mg/dL. Additionally, the ORs for kidney stones in patients with HbA1c levels of 5.5 to 6.0%, 6.0 to 6.5%, and ≥6.5% were 1.16 (95% CI: 0.76–1.79), 1.25 (95% CI: 0.70–2.23), and 1.98 (95% CI: 1.11–3.52), respectively, compared to those in patients with HbA1c level <5.5%.^[23]^

In our preliminary analyses, the HbA1c level was significantly higher in UA stone formers than in nonUA stone formers. The HbA1c level may reflect glycemic control in patients with diabetes. We hypothesize that this result may be attributable to a low urinary pH level, which is inversely related to HbA1c level. In their previous study, Torricelli et al showed a significant negative correlation between HbA1c and urinary pH levels.^[24]^ Therefore, adequate glycemic control by dietary regulation and medication use, if needed, may decrease the incidence or recurrence of UA stones.

Several studies on stone analysis have found that the incidence of UA stones increases with age.^[13,25]^ A population-based study by the Mayo Clinic^[14]^ reported similar results. Aging may be associated with decreased renal ammoniagenesis and increased urine acidity, promoting urate supersaturation. These reports are consistent with our findings that age is a risk factor for urolithiasis, particularly UA stones.

Urinary stones develop more commonly in men than in women, with an estimated male-to-female ratio ranging from 1.7:1 to 3:1.^[13,14]^ Dietary habits play an important role in the development of urinary stones. Males tend to consume a high-protein diet compared to females, thus resulting in increased excretion of calcium, oxalate, and UA in the urine. This is followed by oversaturation of urate in the urine, resulting in the development of UA stones.^[26]^ Our results were similar to previous studies, suggesting that UA stones occurred more frequently in males than in females.

Additionally, in this study, UA stones developed more frequently in the bladder than in the kidney in comparison to nonUA stones. The causes of bladder stones include nutritional deficiency, infection, and lower urinary tract obstruction.^[27]^ These conditions can induce excessively acidic urine pH, hyperuricosuria, and hypocitraturia. This biochemical alteration is a significant risk factor for UA stone formation.^[10]^ We observed no difference in stone composition between kidney and ureteral stones.

Our study has several limitations. First, data on the 24-hour urine composition was unavailable or unobtainable in many cases due to this study’s retrospective nature. Furthermore, several patients refused to undergo the test because of the considerable time required to obtain urine samples. However, we believe that urinary stone composition is a more meaningful clinical endpoint than urine composition for this study; therefore, we did not perform urine composition analysis. Nevertheless, accumulated data on urine composition would be required for more accurate analysis in the future. Second, although urinary pH may be affected by an individual’s activity level, measurement hour, and diet, this could not be considered owing to marked individual variations. Lastly, we could not evaluate the effects of antidiabetic medication type (i.e., insulin or oral antihyperglycemic agents) because many patients were treated with multiple combinations of medications for DM to improve their glycemic control; therefore, a survey on this factor may help identify the relationship between medication type and stone recurrence.

## 5. Conclusions

Age and DM were independent risk factors for UA urolithiasis, implying a relationship between urinary stone composition and metabolic diseases. Furthermore, renal function and HbA1c level were risk factors for UA stones. It is expected that controlling metabolic diseases and preserving renal function could help prevent UA stone formation. Moreover, HbA1c levels are known to reflect the metabolic status of patients with DM. Therefore, adequate management of blood glucose levels may have a preventive effect against UA stones. Further studies are required to explore the association between metabolic risk factors and urolithiasis to investigate the underlying pathways that result in their strong association and formulate suitable management protocols in a large population.

## Author contributions

Conceptualization and Data curation: J.Y. Kim and S.G. Kang

Formal analysis: J.H. Yu and S.H. Kang

Methodology: J.G. Lee, and J. Cheon

Supervision: S.G. Kang

Writing – original draft and Writing – review & editing: J.Y. Kim

## Acknowledgment

The authors would like to thank all our participants for their gracious participation in this study.

## Supplementary Material


