# Supplementary material for: The effect of metabolic risk factors on urinary stone composition: An observational study
Source: Medicine (Baltimore). 2022 Jul 15;101(28):e29622. doi: 10.1097/MD.0000000000029622 (PMC11132329; doi:10.1097/MD.0000000000029622)
Supplement: Supplementary file 1 [file medi-101-e29622-s001.pdf]

Supplementary Table 1. Results of the comparison of parameters, including underlying diseases and laboratory results, according to urinary stone composition.

	UA [a]	CaOx [b]	Carbapatite [c]	Struvite [d]	Brushite [e]	Cysteine [f]	P-value
Patient (n)	137 (23.5%)	340 (58.3%)	69 (11.8%)	21 (3.6%)	9 (1.5%)	7 (1.2%)	
Male/female (n)	113/24 (82.5/17.5%)	218/122 (64.1/35.9%)	41/28 (59.4/40.6%)	9/12 (42.9/57.1%)	7/2 (77.8/22.2%)	6/1 (85.7/14.3%)	**<.001**
Age	65.5±11.96 [bcef]	56.8±13.35 [af]	54.6±15.25 [af]	60.4±13.64 [f]	52.6±17.50 [a]	41.0±8.50 [abcd]	**<.001**
Underlying disease (n)							
DM	41 (29.9%)	68 (20.0%)	5 (7.2%)	3 (14.3%)	0 (0%)	0 (0%)	**.001**
HTN	71 (51.8%)	148 (43.5%)	15 (21.7%)	10 (47.6%)	2 (22.2%)	1 (14.3%)	**.002**
CKD	20 (14.6%)	14 (4.1%)	1 (1.4%)	2 (9.5%)	0 (0%)	0 (0%)	**.001**
CAD	18 (13.1%)	24 (7.1%)	3 (4.3%)	1 (4.8%)	0 (0%)	0 (0%)	.137

Laboratory test							
eGFR	62.7±30.03[bcde]	91.4±30.50[a]	99.5±33.32[a]	86.9±35.60[a]	97.1±37.17[a]	87.9±26.15	**<.001**
Calcium	9.0±0.66[b]	9.2±0.59[ac]	9.0±0.72[b]	9.1±0.68	9.5±0.96	9.3±0.63	**.002**
Glucose	135±73.14[bc]	119±41.44[a]	113.1±24.79[a]	110±17.99	93.4±12.10	105±19.92	**.004**
HbA1c	6.7±1.29[b]	6.2±1.21[a]	5.8±0.94	6.0±1.14	5.4±0.10	5.1±0.28	**.017**
UA	6.7±2.03[bcd]	5.5±1.55[a]	5.0±1.55[a]	5.5±2.30[a]	5.6±2.58	5.6±1.22	**<.001**
Urine pH	5.6±0.69[bcd]	6.0±0.91[a]	6.2±0.89[a]	6.3±1.06[a]	5.9±0.78	6.5±0.58	**<.001**

Using the chi-square test, + ANOVA test. Post hoc analysis was performed using the Student-Newman-Keuls test.

CAD, coronary artery disease; CaOx, calcium oxalate; CKD, chronic kidney disease; DM, diabetes mellitus; eGFR, estimated glomerular filtration rate; HbA1c, glycosylated hemoglobin; HTN, hypertension; UA, uric acid.
